# First report of Tasmanian sheep strain (G2) genotype isolated from Iranian goat using the high resolution melting (HRM) analysis

**Published:** 2016-12

**Authors:** Ahmad Hosseini-Safa, Mohammad Ali Mohag, hegh, Nader Pestechian, Maryam Ganji, Rasoul Mohammadi, Reza Mahmoudi Lamouki, Mohammad Rostami-Nejad

**Affiliations:** 1 Department of Medical Parasitology and Mycology, School of Public Health, Tehran university of Medical Sciences, Tehran, Iran; 2 *Department of Parasitology and Mycology, School of Medicine, Isfahan University of Medical Science, Isfahan, Iran*; 3 *Department of Immunology, School of Medicine, Isfahan University of Medical Sciences, Isfahan, Iran*; 4 *Proteomics Research Center, Shahid Beheshti University of Medical Sciences, Tehran, Iran*; 5 *Gastroenterology and Liver Diseases Research Center, Research Institute for Gastroenterology and Liver Diseases Shahid Beheshti University of Medical Sciences, Tehran, Iran*

**Keywords:** *Echinococcus granulosus*, G2 genotype, HRM, goat, Iran

## Abstract

**Aim:**

The present study was aimed to evaluate *E. granulosus *genotypes isolated from goats using HRM analysis in Isfahan province.

**Background:**

Cystic echincoccosis, so-called hydatidosis, is widespread infection caused by the larval stage of *Echinococcus granulosus*. This is an important zoonotic disease worldwide, especially in the developing countries such as Iran. To date, molecular studies mainly based on the mitochondrial DNA sequences have identified distinct genotypes termed G1-G10 which can differ in some characteristics such as the growth and infectivity to different intermediate hosts or the survival rate in the definitive hosts that are important for the development of control strategies.

**Methods:**

From August to December 2014, 1341 goats were investigated and hydatid cysts were collected from the liver and lungs of 43 infected goats in Isfahan province abattoirs, Isfahan, Iran. Total genomic DNA was extracted from each sample, amplified for the presence of polymorphism of mitochondrial gene coding for cytochrome c oxidase subunit 1 (CO1), using high resolution melting curve (HRM) method.

**Results:**

the results of HRM analysis using the sequence of CO1 gene for 43 *Echinococcus granulosus *isolates from goats showed 31, 2 and 10 isolates were identified as G1, G2, and G3 genotypes, respectively.

**Conclusion:**

G1 is the predominant genotype in the isolated goat samples in Isfahan province, and the presence of G2 strain was reported for the first time in goat in Iran.

## Introduction

Infection of humans and animals with the cestode *Echinococcus granulosus *(*E. granulosus*), so-called hydatidosis, is one of the most important and prevalent parasitic diseases in different parts of Iran (1-3). *E. granulosus *in the domestic animals is detected only at the time of post-mortem inspection at the slaughterhouse. It can cause economic losses in livestock as well as high mortality in humans (4,5). The prevalence of adult worms has been reported from dogs, wolves and jackals in the Middle East and Iran (6). In addition to humans, sheep, goats, buffaloes, camels, horses, cattle and pigs are as intermediate hosts of *Echinococcus *spp. Metacestode stage of the parasite is routinely found in the viscera of the mentioned animals, especially in their liver and lung (1,7). *E. granulosus *shows a wide range of intra-specific variation related to host specificity, biology, morphology, epidemiology and genetics (8). To date, molecular studies mainly based on the mitochondrial DNA sequences have identified 10 distinct genotypes termed G1- G10 (9,10). Different genotypes have been known as sheep strain (G1), Tasmanian sheep strain (G2), buffalo strain (G3), horse strain (G4), cattle strain (G5), camel strain (G6), pig strain (G7), cervid strain (G8), human polish strain (G9), and Fennoscanadian cervid strains (G10) (11). All defined genotypes were divided into five species:

*E. granulosus sensustricto *(G1-G3), *E. equinus *(G4), *E. ortleppi *(G5), *E. canadensis *(G6, G7, G8 and G10) and *E. felides *(G9) (12,13). These genotypes can differ in some characteristics such as the growth and infectivity to different intermediate hosts or the survival rate in the definitive hosts that are important for the development of control strategies (14).

The Iranian Veterinary Organization (IVO) reported that the population of goats is estimated to be 25,800,000 (15). The prevalence of hydatid cysts in goats has been reported in Iran from 1.7 to 29.4% (16-19). Molecular identification using nested PCR, PCR-RFLP and real-time PCR has widely been used for the detection of *E. granulosus *genetic variations (20,21). The present study was aimed to evaluate the *E. granulosus *genotypes in the samples isolated from goats using high resolution melting curve (HRM) analysis in Isfahan province located in the center of Iran.

## Materials and Methods

**Sample collection**

From August to December 2014, 1341 goats were examined and hydatid cysts were collected from the liver and lungs of 43 infected goats in Isfahan province abattoirs, Iran. Out of 43 samples infected with hydatid cyst, 37 were fertile and 7 were infertile. All hydatid cysts were obtained under sterile condition, and then protoscolices and/or the germinal layer were collected from an individual hydatid cyst. In order to perform molecular analysis, the protoscolices and germinal was performed using Mini Opticon real-time PCR detection system (Rotor-Gene 6000, Germany) in a volume of 25 µL containing 10 µLmaster mix (HRM PCR Kit, Qiagen, 100 Germany), 10 µL distilled water, 1 µL each primer with 10 pmol/ul concentration volume and 4 µL template DNA under the following thermal profile: 10 min at 95 °C for initial denaturation followed by 40 cycles at 95°C for 10s for denaturation, 55 °C for 15 s for annealing and 72 °C for 27 s for extension and a final extension step at 72°C for 5 min. After an initial step of 95 °C for 3 min and 40 °C for 1 min, melting curve was obtained by increasing the temperature from 65°C to 85°C at intervals of 0.2 °C per 2s.

**DNA sequencing and phylogenetic analysis**

To confirm the identified genotypes, 6 samples of different curve were randomly sequenced for cox1. The obtained sequences were compared with previously published sequences of the mitochondrial CO1 gene for *E. granulosus *genotypes in NCBI using basic local alignment search tool (BLAST) system. Phylogenetic analyses of the sequence data were inferred with maximum likelihood using the Molecular Evolutionary Genetics Analysis (Mega5) software package (version 5.2.1, 2013) (23-25).

## Results

**Figure 1:**
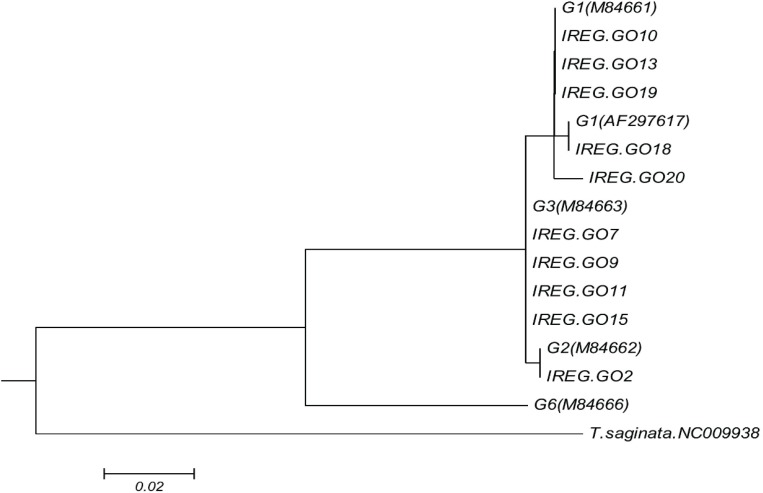
Molecular phylogenetic tree of 10 *E. granulosus *isolates of goat along with reference isolates based on CO1 gene sequence. The evolutionary history was inferred by using the Maximum Likelihood method based on the Kimura 2 parameter model (23). The tree with the highest log likelihood (-814.2241) is shown. Reference accession nos.: G1, M64661; G2, M64662; G3, M64663; G6, M84666

**Figure 2:**
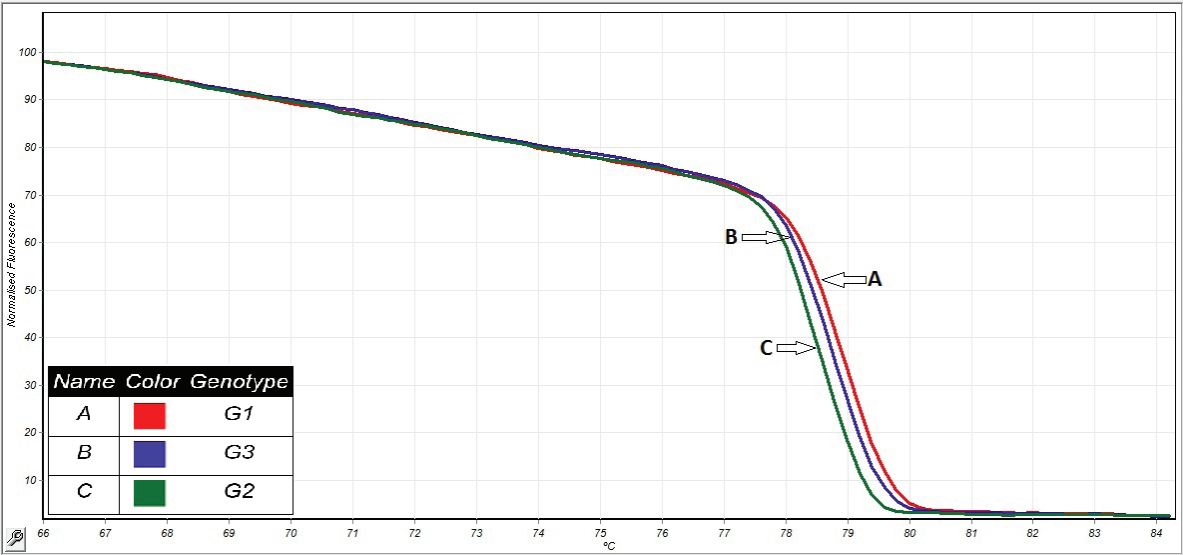
HRM based on (EVA Green-TM) curve analyses of *E.* *granulosus* identified by sequencing (A-C). (A) G1, (B) G3, and (C) G2 genotype

Out of 43 hydatid cysts isolated, 28 of them were collected from liver and 15 from lungs; 37 cysts were fertile and 7 infertile. All the isolates identified by HRM were clustered along with the corresponding reference genotypes as shown in Figure 1. HRM analysis using the sequence of CO1 gene for 43 *E. granulosus *isolates from goats showed G1 genotype was identified in 31 and G3 in 10 isolates. For the first time G2 genotype was detected in the 2 collected isolates (Figure 2).

layer were stored in 70% ethanol at -20 ˚C until DNA Also, T analysis was used for the detection of *E. granulosus *extraction.

**DNA extraction**

The samples were rinsed three times with sterile distilled water to remove the ethanol prior to DNA extraction. Total genomic DNA was extracted using the genomic DNA extraction kit (Bioneer, Daejeon, Korea) according to the manufacturer’s instructions with some modifications. Concentration of DNA was determined by Nano Drop and then samples were stored at -20 ˚C until DNA amplification.

**DNA amplification and melting curve (HRM) analysis**

**Figure 3:**
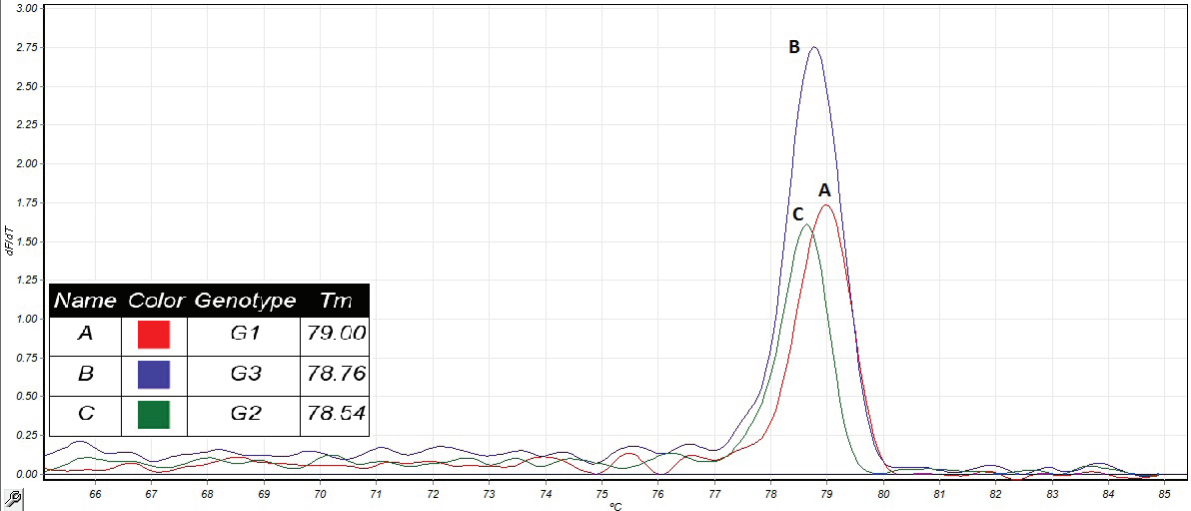
T_m_ of the analyzed hydatid cysts. (A) *E. granulosus* G1 identified by sequencing, (B) *E. granulosus* G3 identified by sequencing, and (C) E. granulosus G2 identified by sequencing.

The template for amplification was the mitochondrial sequences coding for cytochrome c oxidase subunit 1 (CO1) gene. The amplification was carried out by real- time PCR using the following primers: forward JB3 (5´-TTTTTTGGGCATCCTGAGGTTTAT-3´) and reverse JB4.5 (5´-TAAAGAAAGAACATAATGAAAATG-3´)

described by Bowles et al. in 1992 (22). The PCR reaction genotypes (G1, G2 and G3 genotypes) in goats in Isfahan province, Iran (Figure 3).

## Discussion

HRM is a reliable, less time-consuming and cost-effective technique for identification of helminthic parasites that can be, so effective and beneficial for genotyping *E. granulosus *(26,27). In the present study, the CO1 gene was used to identification of *E. granulosus *genotypes. CO1 gene is one of the best targets to discriminate strains, genotypes and microvariants of *Echinococcus *spp*. *(28,29). A few studies have been done on the genotypes of *E. granulosus *on goats in Iran (15,30). In the present study, molecular results of the samples isolated from goats demonstrated the presence of three *E. granulosus *strains, including the common sheep strain (G1), Tasmanian sheep strain (G2) and buffalo strain (G3). We isolated G2 strain by using HRM analysis for the first time on livestock in Iran. The G2 genotype occurred in two liver samples of goats. In two previous studies, G2 strain was isolated from human and dog in Iran (31,32). In the other countries, the G2 strain was isolated from cattle in Italy and sheep in Argentina (33,34). Out of 43 samples from goats, 31 isolates (72.1%) were G1 genotype (sheep strain). The G1 genotype is the dominant strain in both human and animals in Iran and the world (35).

In all studies carried out on goats in Iran, G1 genotype was isolated (36) that is evidence for the goat is a good intermediate host for the sheep strain. In the present study, G3 genotype (buffalo strain) was isolated from 10 (23.2%). G3 genotype has been reported in the intermediate hosts including sheep, goat, cattle, pigs, and human, and definitive hosts in Iran and other countries such as India, Turkey, Pakistan, Italy and Greece (10,37). In a similar study on goats in Isfahan, G3 genotype in 25% of cases was observed using CO1 fragment (38), but in studies on the hydatid cysts isolated from goats in Mazandaran and Lorestan provinces, as well as Varamin city in Iran, G3 genotype was not found (30,36). These findings indicate that the goats can be important intermediate host for of the analyzed hydatid cysts. (A) *E. granulosus *G1 identified by sequencing, (B) *E. granulosus *G3 identified by sequencing, and (C) *E. granulosus *G2 identified by sequencing.

In conclusion, HRM analysis is a reliable and rapid technique for screening and discrimination of different species and genotypes within *E. granulosus*. G1 is predominant genotype in goats in Isfahan province, but the presence of G2 strain was detected for the first time in livestock in this area and should be noted in the intermediate hosts.
